# Supplementary material for: Splice-Junction-Based Mapping of Alternative Isoforms in the Human Proteome
Source: Cell Rep. Author manuscript; Available in PMC 2020 Jan 15. (PMC6961840; doi:10.1016/j.celrep.2019.11.026)
Supplement: 2 [file NIHMS1546469-supplement-2.zip › DF1/PXD009737/TestisTrypsin/TestisTrypsin_44_UBXN11_PGPGPSPGPGPGPSPGPGPGPSPCPGPSPSPEAR.pdf]

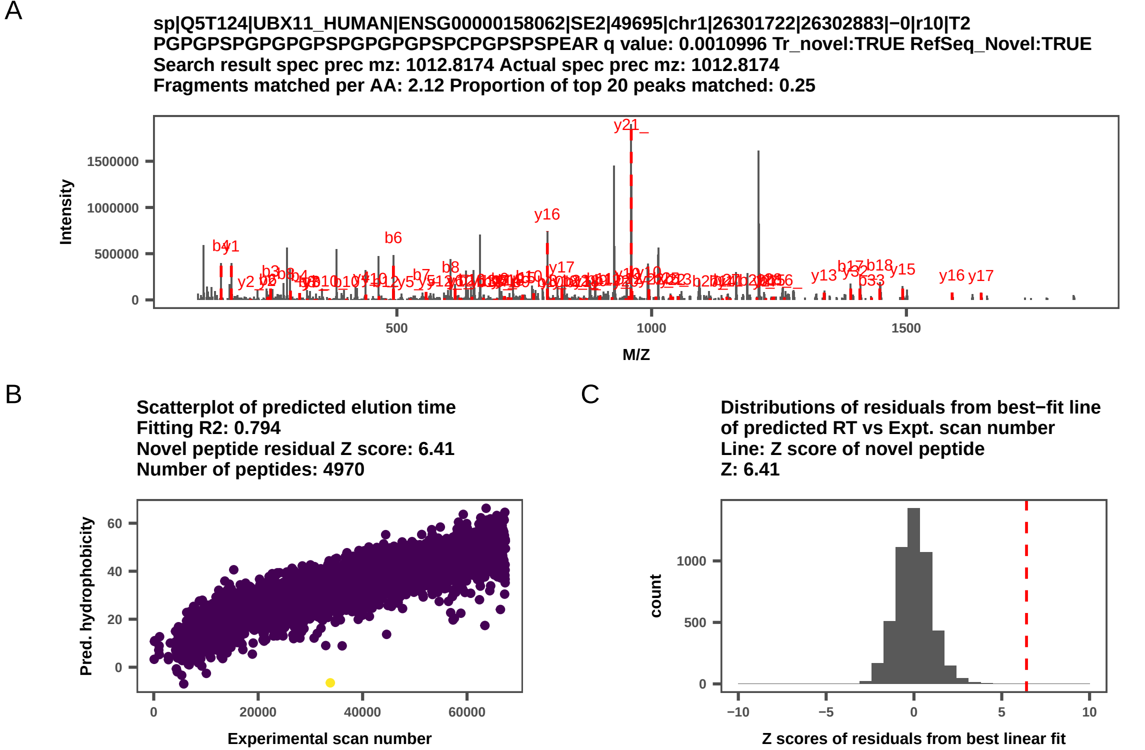
A
sp|Q5T124|UBX11_HUMAN|ENSG00000158062|SE2|49695|chr1|26301722|26302883|−0|r10|T2
PGPGPSPGPGPGPSPGPGPGPSPCPGPSPSPEAR q value: 0.0010996 Tr_novel:TRUE RefSeq_Novel:TRUE
Search result spec prec mz: 1012.8174 Actual spec prec mz: 1012.8174
Fragments matched per AA: 2.12 Proportion of top 20 peaks matched: 0.25
Intensity
1500000
1000000
500000
0
500
1000
1500
M/Z
y21_
y16
b6
b8
y17
y15
y13
y16
y17
B
Scatterplot of predicted elution time
Fitting R2: 0.794
Novel peptide residual Z score: 6.41
Number of peptides: 4970
Pred. hydrophobicity
60
40
20
0
0
20000
40000
60000
Experimental scan number
C
Distributions of residuals from best−fit line
of predicted RT vs Expt. scan number
Line: Z score of novel peptide
Z: 6.41
count
1000
500
0
−10
−5
0
5
10
Z scores of residuals from best linear fit
